# Framing Childhood Anemia as an Adverse Childhood Experiences-Associated Health Condition: An Opportunity to Support Healthy Growth and Development

**DOI:** 10.7759/cureus.93505

**Published:** 2025-09-29

**Authors:** Beckett Z Hutchens, Joseph P Benitez, Nina Thompson, Kelsi E McCoy-Wilson, Randall Chan, Amy J Shekarchi

**Affiliations:** 1 Department of Pediatrics, University of California Los Angeles David Geffen School of Medicine, Los Angeles, USA; 2 ACEs-LA Innovation Lab, Olive View University of California Los Angeles Medical Center, Sylmar, USA; 3 ACEs-LA, Los Angeles County Department of Health Services, Los Angeles, USA; 4 Department of Pediatrics, Olive View University of California Los Angeles Medical Center, Sylmar, USA; 5 Department of Pediatrics, University of Southern California Keck School of Medicine, Los Angeles, USA

**Keywords:** ace-associated health condition, adverse childhood experience, adverse childhood experiences (aces), childhood anemia, food insecurity, iron deficiency, pediatric preventive care

## Abstract

Introduction: Both childhood adversity and childhood anemia have the potential to impact growth and development among young children. This study examined the relationship between Adverse Childhood Experience (ACE) scores and hemoglobin levels in young children receiving care in a safety net health system in California.

Methods: We conducted a retrospective review of electronic health records for pediatric patients ages zero to five years who received both ACE screening and hemoglobin testing between 2020 and 2025. Hemoglobin results were categorized as severe anemia (<7.0 mg/dl), mild-moderate anemia (7.0-10.9 mg/dl), and normal (>11.0 mg/dl). ACE scores were categorized into Low-Intermediate Risk (ACE score 0-3) and High Risk (ACE score ≥4).

Results: We found an association between higher ACEs and lower hemoglobin levels across all patients and within the subset of patients with mild-moderate anemia. Among a population of 8,979, 21.7% had any ACE, 18.3% reported ACE scores of 1-3, and 3.4% had ACE scores of ≥4. Mean hemoglobin was lower in the ACEs High Risk group compared to the ACEs Low-Intermediate Risk group (12.2 mg/dl vs. 12.6 mg/dl, p < 0.001). In the subgroup with mild-moderate anemia (N = 716), ACEs High Risk children also had lower mean hemoglobin levels (9.9 mg/dl vs. 10.3 mg/dl, p < 0.05). Moreover, linear regression models showed that each one-point increase in ACE score resulted in a 0.078 mg/dl decrease in mean hemoglobin (p < 0.0001).

Discussion: While statistically significant, the 0.4 mg/dl difference in hemoglobin levels alone does not often have clinical implications for treatment. However, in the context of high ACE scores, this difference may help guide preventative and treatment efforts.

Conclusions: The association of higher ACE scores with lower hemoglobin levels in young children highlights an opportunity to better identify factors that may contribute to anemia in pediatric patients. Knowledge of such risk factors can inform efforts to provide comprehensive counseling and support for patients at risk for anemia. Further research will help determine whether integrating ACE screening into anemia prevention strategies could impact developmental and health outcomes in high-risk populations, and this inquiry may further establish childhood anemia as a meaningful ACE-associated health condition that can be identified and addressed before long-term health consequences emerge.

## Introduction

Health care providers caring for children from low-income households face many challenges assessing health status and addressing social factors that impact both short- and long-term health outcomes. During pediatric visits, providers assess growth and nutritional status, provide vaccines, screen for food insecurity, check for achievement of developmental milestones, review home safety, and use multiple preventive health strategies to optimize health [1]. With limited time for visits, it is challenging to determine which of these areas to prioritize.

Screening for Adverse Childhood Experiences (ACEs) has become a valuable source of information for pediatric providers as they engage with children and families during primary care visits. ACEs are traumatic events that happen before the age of 18 that are associated with poor health outcomes in adulthood [2,3]. ACE-associated health conditions range from medical conditions such as cancer and heart disease to mental health conditions [2].

ACEs are common, impacting 63.9% [4] of adults and 46.3% of children in the United States [5]. A recent meta-analysis found that 7.6% of children ages zero through 12 have experienced 4 or more ACEs [6]. ACEs are more common in low-income and minoritized populations, and another study identified that 4.3% of U.S. children who reside in low-income households report ACE scores of 4 or more [7]. Financial hardship, parental stress, limited resource access, and other social challenges that impact low-income or marginalized communities may contribute to their increased ACE risk.

In children, ACE screening can be conducted using the Pediatric ACEs and Related Life Events Screener (PEARLS), which includes an assessment for ACEs as well as other stressful life events [8]. Current investigations are narrowing in on the use of ACE screening and response activities as a tool to help identify and mitigate ACE-related health conditions - including asthma and obesity, which often emerge early in childhood [9]. Iron-deficiency anemia may also be an important area to examine in the context of ACEs, as it has a higher prevalence in low-income communities, is more likely to occur when families face food insecurity, and its identification and treatment have the potential to significantly impact childhood health and development [10, 11].

Similar to ACEs, iron-deficiency anemia is more common in lower-resourced settings due to food insecurity, prolonged exclusive breastfeeding associated with delayed food introduction, and limited health care access [12]. As such, the Women, Infants, and Children (WIC) Program and CDC recommend screening for anemia starting at nine to 12 months of age with follow-up through age six years to ensure that it is identified and addressed [13]. The most common cause of anemia in this age group is iron deficiency [14], which contributes to poor growth, neurocognitive deficits, poor motor outcomes, and other long-term negative health consequences [15-16]. As such, hemoglobin screening among young children is recommended to help identify and treat iron-deficiency anemia and, in turn, support healthy growth and development [17,18].

While iron-deficiency anemia has not been systematically evaluated and/or categorized as an ACE-related health condition, one study noted a higher prevalence of anemia in elderly adults with ACEs [19]. Given the increased likelihood of ACEs and iron-deficiency anemia in children in low-income communities, along with the known negative impacts on health from both ACEs and iron deficiency, we sought to examine the relationship between ACE scores and hemoglobin levels among young children receiving primary care in a large outpatient safety net clinic network in California.

## Materials and methods

This is a retrospective cross-sectional study of pediatric patients receiving primary care in a safety net health system in Los Angeles County, comprised of four hospital-based clinics and 10 comprehensive outpatient health centers. We accessed data available in the electronic health record in June of 2025 to review ACE scores and hemoglobin results, as well as demographic data related to primary care visits for children aged zero to five years between January 1, 2020, and May 31, 2025. This date range coincides with the initiation of ACE screening in our health system and ends in the month prior to initiation of the analysis.

Children in our health system receive ACE screening at their initial primary care visit and once yearly thereafter, usually during a well child visit. During the screening process, parents receive the Pediatric ACEs and Related Life Events Screener (PEARLS) from a clerk or nurse at check-in. Their completed questionnaire is reviewed by and discussed with the provider during the child's visit.

ACE scores were extracted from the entry of Part 1 of the PEARLS in the electronic medical record. When more than one score was recorded for a single patient, the highest score during the review period was used for this analysis. ACE scores were categorized by number and were also grouped into 0-3 (Low-Intermediate Risk) and 4+ (High Risk) in alignment with the ACEs Aware categorization of ACE scores [9].

Serum hemoglobin levels were also extracted from the electronic medical record from lab collections completed as part of the patients’ usual care. When more than one hemoglobin was recorded during the review period, the lowest hemoglobin value was used for this analysis. Because ACE screening and hemoglobin screening do not consistently occur at the same visit, defining and selecting hemoglobin levels taken in proximity to ACE screening was not possible.

Hemoglobin results were divided first using the norms for the lab in our health system: <10.9 mg/dl (low); 11-14.9 mg/dl (normal range); and 15 mg/dl or greater (above normal range) and then as follows for anemia classification according to published reference ranges from the World Health Organization for children ages zero to five years of age: <7.0 mg/dl (severe anemia); 7-10.9 mg/dl (mild-moderate anemia); 11 mg/dl or greater (no anemia) [20].

Because routine screening for hemoglobin to help identify iron deficiency anemia is not recommended prior to age nine months, hemoglobin results posted prior to nine months of age were not considered in the analysis. In addition, any child with more than five reported hemoglobin results was excluded from the study population to avoid inclusion of test results possibly associated with acute or chronic medical conditions. Among children who posted hemoglobin levels of <7 mg/dl, a chart review was conducted to rule out anemia due to a pathologic process such as severe infection or leukemia; none were identified. Similarly, among children whose hemoglobin measurements were 15 mg/dl or above, a chart review was conducted to rule out pathologic polycythemia; none were identified.

Pediatric patients aged zero to five years who underwent both ACE screening and serum hemoglobin (after the age of nine months) between January 1, 2020, and May 31, 2025, were included in the study sample.

Statistical analysis was performed using odds ratios, mean comparison tests, and linear regression. Statistical significance was defined with p < 0.05. Tables were prepared using STATA (Version 18.5, StataCorp LLC, College Station, TX, United States). Analysis focused on the following: 1) hemoglobin levels and ACE risk stratification for the entire study population, and 2) mild-moderate anemia (hemoglobin 7-10.9 mg/dl) and ACE risk stratification. This subgroup was chosen because it represents the most common anemia classification treated by primary care providers and because this group offered a sample size conducive to meaningful analysis.

All activities were performed after approval from the affiliated institutional review board and in accordance with the Declaration of Helsinki.

## Results

During the review period, 8,979 children had a documented ACE screen obtained between zero and five years of age, and a hemoglobin lab result obtained between nine months and five years of age. This sample accounts for approximately 73% of all patients aged zero to five years who received primary care in the health systems’ clinics during the study period.

Characteristics of the study population can be found in Table 1. More than 90% were enrolled in or qualified for Medi-Cal (California’s Medicaid program), about 52% were male, and the majority were Hispanic/Latino or African American. Sex and race/ethnicity among the study population were not significantly different from the overall primary care population in participating clinics. In our health system, patient sex and race/ethnicity are defined by patients or their legal caregivers during visit check-in and are recorded in the patient’s electronic health record.

**Table 1:** Children aged zero- to five years with ACE screen and hemoglobin results (N = 8,979) ACE: Adverse Childhood Experience

Characteristics	Study Sample
Age	0-5 years
Gender	
Male	4,663 (51.9%)
Female	4,316 (48.1%)
Race/Ethnicity	
Hispanic/Latino	5,569 (62.0%)
Black/African American	1,071 (11.9%)
Asian/Pacific Islander	304 (3.4%)
American Indian/Alaska Native	19 (0.2%)
White	174 (1.9%)
Other	1,842 (20.5%)
Insurance Status	
Medicaid	8,003 (89.1%)
Other	900 (10.0%)
Medicaid eligible	76 (0.9%)
ACE Score	
0	7,034 (78.3%)
1 to 3	1,640 (18.3%)
4+	305 (3.4%)
Hemoglobin Levels	
<7 (severe anemia; no underlying disease process)	7 (0.08%)
7-10.9 (mild anemia)	716 (7.97)
11-14.9 (normal)	7,780 (86.65%)
15 or greater (no underlying disease process)	476 (5.30%)

ACE scores ranged from 0 to 10, with the majority reporting ACE scores of 0; 18.3% reporting 1-3 ACEs; and 3.4% reporting 4+ ACEs. ACE scores for the study population were not statistically significantly different from those found among the overall primary care population in participating clinics.

**Figure 1:**
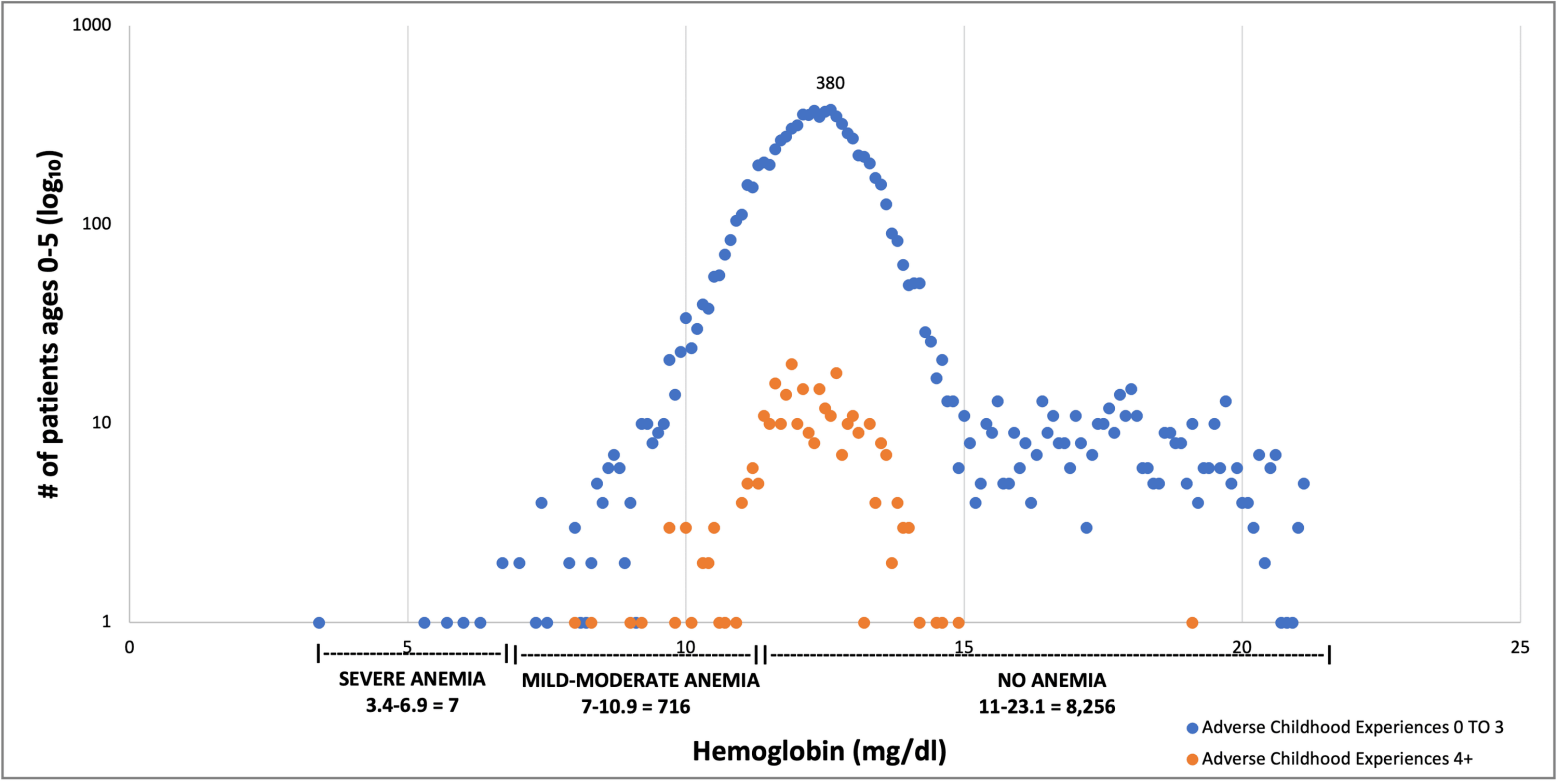
Scatterplot of hemoglobin values and ACE scores (N = 8,979) ACE: Adverse Childhood Experience

The distribution of ACE scores and hemoglobin results for the study population is shown in Figure 1.

Hemoglobin levels and ACE risk stratification

To examine the relationship between hemoglobin levels and ACEs risk status, a T-test was used to compare mean hemoglobin among those with Low-Intermediate Risk (0-3) ACE scores and those categorized as High Risk (4+).

**Figure 2:**
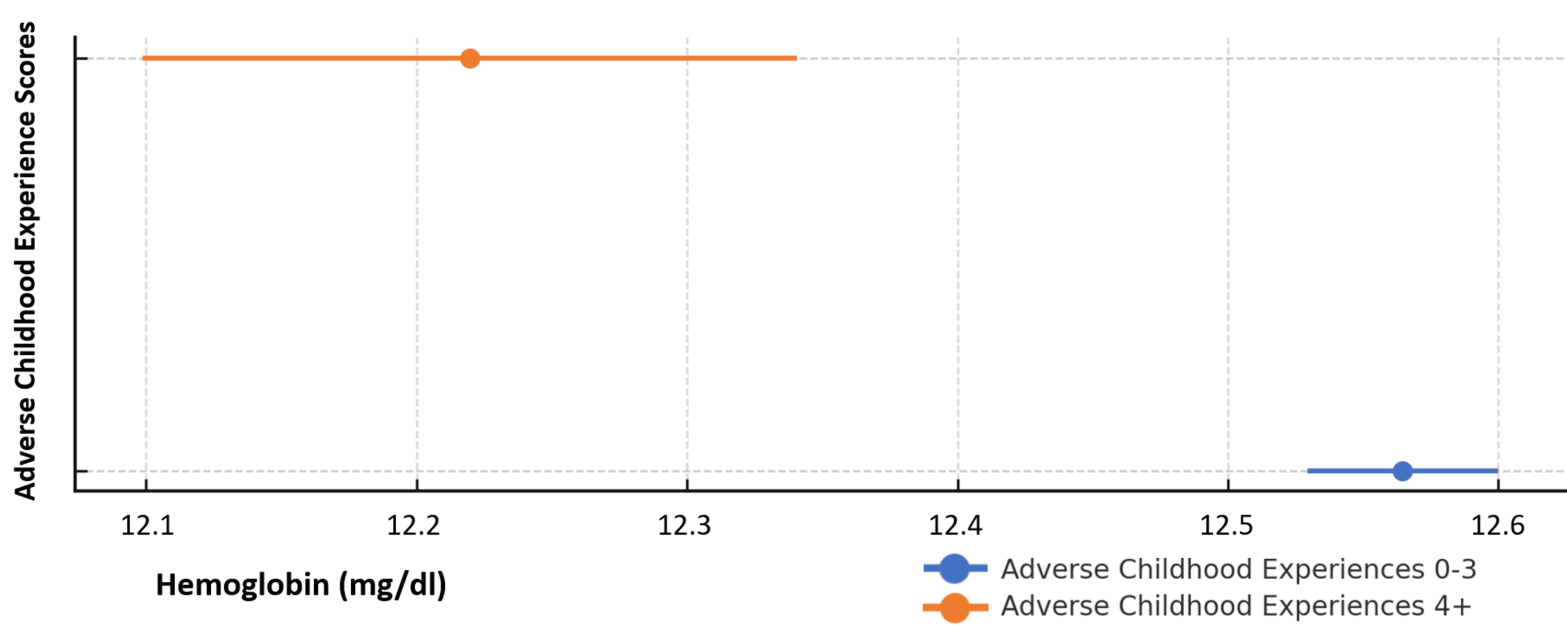
Mean hemoglobin and ACE risk status among all children ages 0-5 years (N = 8,979) Children in the Low-Intermediate risk ACE category had higher mean hemoglobin than those in the High Risk category (12.6 mg/dL vs. 12.2 mg/dL; p < 0.001). ACE: Adverse Childhood Experience

Across the study population, mean hemoglobin levels were observed to be lower among children with ACEs in the High Risk (4+) category compared to those in the Low-Intermediate risk (0-3) category. The difference in mean hemoglobin between those in ACEs Low-Intermediate risk and ACEs High Risk categories (12.6 mg/dl vs. 12.2 mg/dl) was statistically significant (p < 0.001). These differences can be seen in Figure 2 and were not attributable to sex, age, or race.

**Figure 3:**
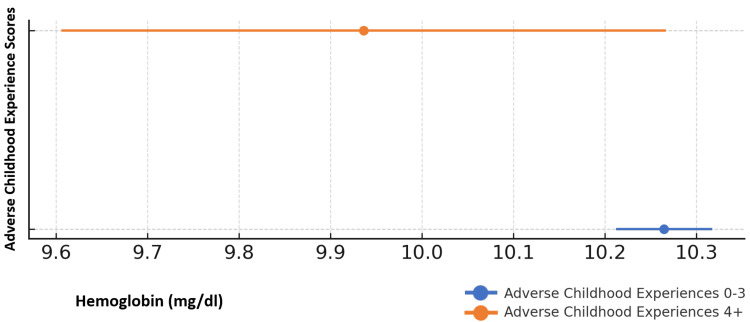
Mean hemoglobin and ACE risk status among children ages 0-5 years with mild-moderate anemia (N = 716) Within the subset of mild-moderate anemia, children in the High Risk ACE category had lower hemoglobin than those in the Low-Intermediate risk group (9.9 mg/dL vs. 10.3 mg/dL; p < 0.05). ACE: Adverse Childhood Experience

Additionally, within the subset of children with mild-moderate anemia (Hgb 7-10.9 mg/dl), those with High Risk (4+) ACE scores also had significantly lower hemoglobin levels (10.3 mg/dl vs. 9.9 mg/dl; p < 0.05), as shown in Figure 3. The subset in this analysis was too small to examine for differences across age, sex, or race.

Hemoglobin regression analysis

Linear regression analysis was applied to the study population to determine trends in hemoglobin values as ACE scores increased. While linear regression presumes that hemoglobin is dependent on ACE score, this analysis was undertaken to explore associations uncovered in the prior analysis of differences in mean hemoglobin levels.

**Figure 4:**
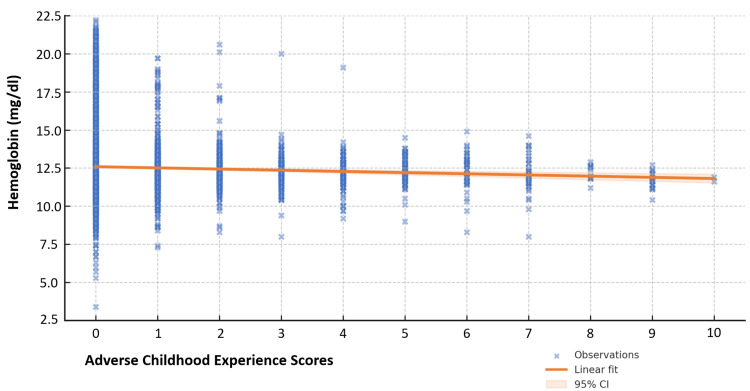
Linear regression shows the hemoglobin trend as ACE scores increase (N = 8,979) ACE: Adverse Childhood Experience

Examining all patients in the test population (N = 8,979), we found that for every one-point increase in ACE scores, mean hemoglobin decreased by 0.078 mg/dl (CI = 95%; p < 0.0001), as shown in Figure 4.

## Discussion

This study evaluated the relationship between ACE scores and hemoglobin levels in a large population of children zero- to five years of age receiving care in a public safety net system. The study population represents a diverse group of young children, nearly all of whom qualified for state-funded health insurance based on family income. This population is at particularly high risk for anemia [12] and for ACEs [7], making this inquiry particularly meaningful. To our understanding, this is the first study to investigate the association between ACE exposure and anemia in children using pediatric primary care data.

Close to 8% of our study population had mild-moderate anemia, which is consistent with national reporting on childhood anemia [10]. Within this same group, the prevalence of high ACE scores (3.7%) was consistent with the prevalence across our health system’s population, but lower than other evaluations of U.S children (4.3-7.6%) [6,7]. 

We found statistically different mean hemoglobin levels among all children with 0-3 ACEs (Low-Intermediate Risk) and children with 4+ ACEs (High Risk) (12.6 mg/dl vs. 12.2 mg/dl, p < 0.001). This difference was also noted in children with mild-moderate anemia in the Low-Intermediate and High Risk ACE categories (10.3 mg/dl vs. 9.9 mg/dl, p = 0.0269). While these observed differences may not have meaningful practice implications at this time - the clinical significance of 0.4 mg/dl difference in hemoglobin levels is often minimal - these findings do highlight an opportunity to better identify factors that may contribute to lower hemoglobin values in pediatric patients. Knowledge of such risk factors can inform efforts to provide comprehensive counseling and support for patients at risk for anemia.

The findings from our regression analysis highlight an interesting pattern in the hemoglobin trend as ACE scores increase - showing that for every one-point increase in ACE scores, mean hemoglobin decreased by 0.08 mg/dl (CI = 95%; p < 0.0001). This predictive decrease, while not clinically relevant for lower ACE scores, predicts a hemoglobin difference of nearly 0.8 mg/dl for an ACE score of 10. This change could certainly have clinical implications in a child with moderate anemia. Further, the high level of significance noted suggests that, even if subtle, this finding may be important to examine further.

Together, our observed differences in mean hemoglobin values and the predicted changes in our linear regression model may prompt pediatric providers to consider ACE screening as a tool in anemia risk assessment and treatment decision-making. For example, higher ACE scores may lead clinicians to consider earlier or more frequent anemia screening or to implement more focused nutritional education. Higher ACE scores may also support developing treatment plans that include consideration of toxic stress or other challenges that could impact treatment adherence. 

Due to the high prevalence of both ACEs and anemia among low-income children, identifying and managing both is an important component of primary care. Both ACE screening and hemoglobin screening are relatively easy to obtain, as evidenced in 73% of all patients ages 0-5 receiving both over our study period, and both screens have the potential to trigger interventions that promote health and well-being. Conducting this study in a population with a relatively high prevalence of both anemia and ACEs provided the advantage of a larger pool of children with anemia and high ACEs, which may be helpful for providers in settings where low prevalence would prevent identification of this association.

Screening for ACEs is quick, and the ACEs Aware initiative has published algorithms to support a tiered approach to response that includes education, referrals, and reassessment [9]. In addition, California and several other U.S. states provide reimbursement for ACE screening and response activities as a supplement to Medicaid payments to support addressing toxic stress and preventing poor long-term health outcomes [21]. Screening and responding to ACEs in children has the potential to support early intervention, address identified trauma, and prevent further adversity [22]. By addressing ACEs in children, providers can help mitigate the impact of traumatic experiences and prevent toxic stress, which is a severe, prolonged activation of the normal stress response that can lead to long-term physical and mental health conditions [23].

Identification of iron deficiency is also relatively easy; however, treatment efforts have not always proven successful. Despite significant advances in addressing anemia in the 1980s, there has been little progress in reducing its prevalence in young children since that time [24]. Studies in children have reported low rates of anemia prescriptions and lack of follow-up [25,26], while others show that some children who are treated with iron supplementation still have anemia post-treatment [27]. The lack of successful treatment for anemia has the potential to contribute to poor nutritional and neurodevelopmental outcomes [15,16]. In addition to iron supplementation, access to iron-rich foods can prove challenging for under-resourced families who may lack access to these types of foods or face other food insecurity. These treatment challenges highlight potentially overlooked social determinants of health, which, in this case, may prevent successful prevention and treatment of anemia.

The findings from this evaluation highlight the relationship between ACEs and hemoglobin levels and help create a pathway to both the identification of anemia and the initiation of strategies to successfully manage it. As ACE screening becomes a standard of preventive care across the U.S., identifying the correlation between ACE scores and hemoglobin levels in young children zero- to five years of age, may help clinicians determine which pediatric patients are at greater risk for anemia, and how to better treat those who are diagnosed with it.

Limitations 

Despite several statistically significant observations, there are important limitations to consider. First, the data was analyzed retrospectively and relied heavily on electronic health record documentation. Due to this, factors such as iron intake, dietary diversity, prolonged exclusive breastfeeding, other environmental factors, and socioeconomic status beyond insurance type were not included in the analysis, but may influence pediatric hemoglobin levels. Timing for ACE screening and hemoglobin tests was also not standardized because of variability in the timing of ACE screening and hemoglobin screening across our health system. However, we do know that once acquired, ACE scores never decrease, and we chose the lowest hemoglobin recorded over the study period to identify as many instances of anemia as possible. The relatively low number of patients reporting ACE scores of 4+ also limits the ability to generalize such a trend across entire populations and may have limited our ability to identify significance in some of our analyses. The regression analysis requires that the hemoglobin level be a dependent variable to the independent variable of the ACE score. Though there is no established dependency of hemoglobin to the ACE score, we felt that regression analysis to determine trends in hemoglobin as ACE scores increase was a worthwhile exploration given patterns identified in our prior analyses. The high significance of the findings across this population is also important to note. Lastly, it is possible that anemia and ACEs are co-occurring. More data review and analysis are needed to determine clear patterns and correlations. 

## Conclusions

This evaluation across a large pediatric primary care population demonstrates potential opportunities for approaching pediatric preventive care when framing anemia as an ACE-related health condition. We found an association between higher ACEs and lower hemoglobin levels across all patients and within the subset of patients with mild-moderate anemia.

Awareness of this potential association gives clinicians the opportunity to approach ACE screening and anemia screening in two ways. First, clinicians identifying high ACE scores may use this information to proactively initiate measures to prevent iron deficiency (the most common cause of anemia in this age group). Second, providers who identify children with mild-moderate anemia may benefit from considering ACEs and other social determinants of health as they develop treatment plans. Future research should further examine and clarify the correlations between ACE scores and anemia, and explore whether early intervention among children with high ACE scores can improve long-term developmental and health outcomes, which may establish anemia as a meaningful ACE-associated health condition.
